# The natural history of low‐grade dysplasia in Barrett's esophagus and risk factors for progression

**DOI:** 10.1002/jgh3.12625

**Published:** 2021-08-06

**Authors:** Mohamed Hussein, Vinay Sehgal, Sarmed Sami, Paul Bassett, Rami Sweis, David Graham, Andrea Telese, Danielle Morris, Manuel Rodriguez‐Justo, Marnix Jansen, Marco Novelli, Matthew Banks, Laurence B Lovat, Rehan Haidry

**Affiliations:** ^1^ Division of surgery and interventional science University College London (UCL) London UK; ^2^ Department of Gastroenterology University College London Hospital London UK; ^3^ Wellcome/EPSRC Centre for Interventional and Surgical Sciences (WEISS) University College London London UK; ^4^ Statsconsultancy Ltd Amersham UK; ^5^ Department of Histopathology UCL London UK

**Keywords:** Barrett's esophagus, endoscopy, gastroenterology

## Abstract

**Background and Aim:**

Barrett's esophagus is associated with increased risk of esophageal adenocarcinoma. The optimal management of low‐grade dysplasia arising in Barrett's esophagus remains controversial. We performed a retrospective study from a tertiary referral center for Barrett's esophagus neoplasia, to estimate time to progression to high‐grade dysplasia/esophageal adenocarcinoma in patients with confirmed low‐grade dysplasia compared with those with downstaged low‐grade dysplasia from index presentation and referral. We analyzed risk factors for progression.

**Methods:**

We analyzed consecutive patients with low‐grade dysplasia in Barrett's esophagus referred to a single tertiary center (July 2006–October 2018). Biopsies were reviewed by at least two expert pathologists.

**Results:**

One hundred and forty‐seven patients referred with suspected low‐grade dysplasia were included. Forty‐two of 133 (32%) of all external referrals had confirmed low‐grade dysplasia after expert histopathology review. Multivariable analysis showed nodularity at index endoscopy (*P* < 0.05), location of dysplasia (*P* = 0.05), and endoscopic therapy after referral (*P* = 0.09) were associated with progression risk. At 5 years, 59% of patients with confirmed low‐grade dysplasia had not progressed *versus* 74% of patients in the cohort downstaged to non‐dysplastic Barrett's esophagus.

**Conclusion:**

Our data show variability in the diagnosis of low‐grade dysplasia. The cumulative incidence of progression and time to progression varied across subgroups. Confirmed low‐grade dysplasia had a shorter progression time compared with the downstaged group. Nodularity at index endoscopy and multifocal low‐grade dysplasia were significant risk factors for progression. It is important to differentiate these high‐risk subgroups so that decisions on surveillance/endotherapy can be personalized.

## Graphical abstract

Our large retrospective study from a tertiary Barrett's center shows clear variability in the diagnosis of low‐grade dysplasia. Nodularity at index endoscopy and location of low‐grade dysplasia were associated with risk of progression in true low‐grade dysplasia patients. Endoscopic therapy and resources should potentially be more focused in this sub‐cohort of patients.

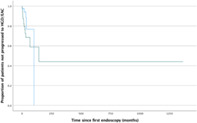

## Background

Barrett's esophagus (BE) is a known risk factor for esophageal adenocarcinoma (EAC), progressing from non‐dysplastic Barrett's esophagus (NDBE), to low‐grade dysplasia (LGD), high‐grade dysplasia (HGD), and then EAC.^1^ EAC is associated with a less than 20% 5‐year survival rate.^2^

Approximately 15–40% of all patients with BE are diagnosed with LGD at some point during their lifetime. LGD has been suggested to be a risk factor for progression to HGD/EAC.^3^, ^4^ Therefore, a clear management strategy for LGD is important. The management of LGD in BE is controversial due to various factors including variability in the natural history of LGD among different populations and interobserver variability among pathologist in its diagnosis.^5^

Variable progression rates have been reported of LGD to HGD/EAC ranging from 0.4 to 13.4%/year.^6^ A randomized study showed a high rate of progression in the surveillance LGD‐BE cohort of patients (26.4% progressed to HGD/EAC).^7^ The diagnosis of LGD was confirmed by an expert pathologist panel. A systematic review showed the cumulative rate of progression to HGD/EAC was lower in the cohort treated with radiofrequency ablation (RFA) *versus* the surveillance group (1.7% *vs* 12.6%, *P* < 0.001).^6^

There has been varying outcomes from studies investigating the natural history of LGD in BE. Specialist Barrett's histopathologists were not involved in many of the studies, which contribute to interobserver variation in the diagnosis of LGD.^8^ There has been variation between studies in regard to risk factors for progression of LGD in BE.^9^, ^10^, ^11^

Table 1 shows some of the main recommendations for management of LGD in BE.^12^, ^13^, ^14^, ^15^, ^16^

**Table 1:** Summary of professional societies' recommendations for management of low‐grade dysplasia‐Barrett's esophagus

Society	Criteria for diagnosis	Follow up	Treatment
American Society for Gastrointestinal Endoscopy (ASGE)^14^ 2012	Confirmation by expert gastrointestinal (GI) pathologist	Repeat endoscopy within 6 months to confirm diagnosis	Consider radiofrequency ablation (RFA) or perform annual surveillance
American Gastroenterological Association (AGA)^13^ 2011	Confirmation by one additional expert GI pathologists	Surveillance every 6–12 months	Consider RFA in confirmed LGD
British society of Gastroenterology (BSG)^15^ 2014	Confirmed by two independent pathologists	Perform endoscopy every 6 months until 2 successive negative diagnosis	Consider RFA
European Society of Gastrointestinal Endoscopy (ESGE)^16^ 2017	Confirmed by a second expert GI pathologist	Repeat endoscopy at 6 months to confirm diagnosis.	Endoscopic ablation offered in confirmed LGD

## Aims

We performed a retrospective study from a tertiary referral center for BE neoplasia to estimate time to progression in patients with confirmed LGD diagnosed by expert histopathologists. The aim of this study was to:

- Compare the risk of progression of confirmed LGD *versus* the cohort of patients downstaged from LGD to indefinite for dysplasia (IND) and NDBE.
- Assess the rates of upstaging/downstaging of LGD following referral.
- Assess the risk factors for progression of confirmed LGD to HGD/EAC.

## Methods

We performed a retrospective cohort analysis of all consecutive BE LGD referrals in a single tertiary center (July 2006–October 2018). All patients underwent high definition white light endoscopy with chromoendoscopy at baseline with targeted and Seattle protocol biopsies following referral. Four quadrant biopsies were taken every 2 cm of the BE segment. All biopsies were reviewed by at least two expert Barrett's histopathologists with more than 10 years of BE pathology experience following which the diagnosis was either downstaged to NDBE/IND, remained the same (confirmed LGD), or upstaged to HGD/EAC. Any confirmed cases of LGD were brought to a multidisciplinary team discussion where a final consensus was reached regarding diagnosis and treatment. Any visible lesions/nodularity at baseline were endoscopically resected, and histology reviewed.

### Definitions

These are the main definitions of outcomes and terms used within the manuscript:

True LGD (T‐LGD): LGD confirmed on index endoscopy following referral and reviewed by two expert Barrett's histopathologists.

Downstaged LGD to NDBE (DS‐LGD‐NDBE): LGD downstaged to NDBE following referral and reviewed by two expert Barrett's histopathologists.

Downstaged LGD to IND (DS‐LGD‐IND): LGD downstaged to IND following referral and reviewed by two expert Barrett's histopathologists.

Unifocal LGD: LGD present on one biopsy level within a segment of BE.

Multifocal LGD: LGD present on more than one biopsy level within a segment of BE.

The inclusion criteria were as follows:

- All patients who meet the standard definition of BE and have LGD.
- Pathology slides reviewed by at least two expert Barrett's histopathologists from index endoscopies following referral.
- Patients did not receive endoscopic eradication therapy prior to referral and had at least one index procedure endoscopy with biopsies at the tertiary center.
- No HGD/EAC in BE histology at index endoscopy following referral and review by two expert BE histopathologists.

Confirmed and eligible LGD patients were offered endoscopic eradication therapy.^15^ A number preferred active surveillance and were monitored. Progression time was defined as the time from the first endoscopy following referral to date of progression to HGD/EAC.

The primary outcome was time to progression to HGD/EAC. Secondary outcomes were risk factors for progression of LGD to HGD/EAC and rates of upstaging/downstaging of LGD following referral to IND or NDBE.

### Statistical analysis

The first analysis summarized the pathological staging of patient following review by an expert histopathologist. Descriptive statistics were used to summarize results.

In patients who were not upstaged time to progression were examined. Survival analysis methods were used. The objective of the analysis was to examine factors associated with the time to progression. The analysis for this outcome was performed using Cox regression. Firstly, the separate association between each factor and the time to progression was examined using univariable analyses. The second stage in the analyses considered the joint association between factors and the outcomes in a multivariable analysis. To restrict the number of variables in this second stage of analysis, only variables showing some association with the outcomes in the univariable analyses (*P* ≤ 0.2) were included.

## Results

A total of 147 patients had a diagnosis of LGD in BE. The median age of patients was 71 (IQR, 64–77) and 86% were male.

One hundred and thirty‐three patients were external tertiary referrals. Forty‐two (32%) of the referred patients had their diagnosis upstaged to HGD following their index endoscopy and review by 2 histopathologists, 49 (37%) patients had their diagnosis downstaged to NDBE (*n* = 31) or IND (*n* = 18), and 42 (32%) patients had the same confirmed LGD diagnosis (Table 2).

**Table 2:** Histology of Barrett's esophagus following expert histopathology review

Patient group	Outcome following expert histology review	Number of patients (%)
All patients (*n* = 147)	Downstaged	49 (33%)
	Same^†^	56 (38%)
	Upstaged	42 (29%)

^†^ Includes 14 non‐referral surveillance patients.

In the confirmed LGD group, a median number of 14 biopsies were taken per patient (Interquartile range, 11–20). In the group downstaged to IND/NDBE, a median number of 14 biopsies were taken per patient (Interquartile range, 8–20).

**Figure 1:**
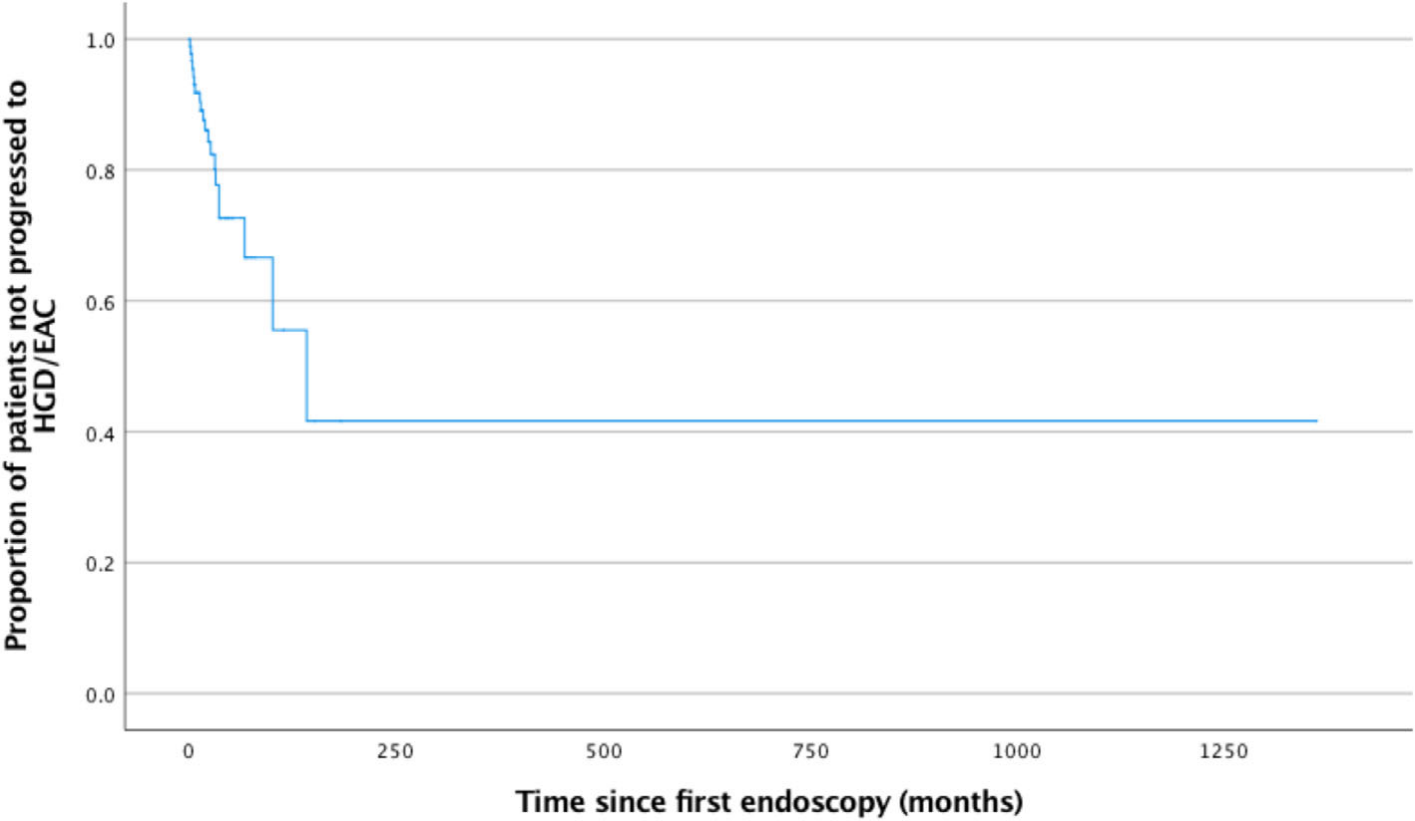
Time to progression to HGD/EAC of all 91 patients who had follow‐up endoscopies with biopsies after their index endoscopy (T‐LGD, DS‐LGD‐NDBE, DS‐LGD‐ID). Time 0 represents the start of the follow‐up period. DS‐LGD‐IND, low‐grade dysplasia downstaged to indefinite for dysplasia; DS‐LGD‐NDBE, low‐grade dysplasia downstaged to non‐dysplastic Barrett's esophagus; EAC, esophageal adenocarcinoma; HGD, high‐grade dysplasia; T‐LGD, true low‐grade dysplasia.

The next analysis examined the time to progression in patients who were not upstaged. For the survival analysis, we omitted the patients who were upstaged at referral (42 patients) and patients who had one index endoscopy at the tertiary center with no follow‐up biopsies (14 patients). This left 91 patients, of these 20 (22%) patients progressed during the follow‐up period (Fig. 1).

Seventy‐three percent of patients had not progressed at 5 years. Fifty‐eight percent had not progressed at 10 years. The median time to progression was 11.8 years (95% confidence interval [CI]: 4.6–19.1 years).

Analysis was performed to examine the factors associated with time to progression in the T‐LGD cohort (*n* = 56) (Table 3). Six patients had no follow‐up endoscopies and were therefore excluded from this part of the analysis. Univariable analysis showed nodularity in BE on index endoscopy, and the location of low‐grade dysplasia (unifocal *vs* multifocal) was significantly associated with time to progression when each factor was considered separately (*P* < 0.05). Patients with nodularity at baseline endoscopy had an increased chance of progression despite endoscopic resection with risk of progression at any time being almost four times greater than patients with no evidence of nodularity on index endoscopy (Hazard ratio 3.56 [1.13, 11.27], *P* = 0.03). Patients with multifocal LGD had an almost five times greater risk of progression compared with patients with unifocal LGD (Hazard ratio 4.82 [1.33, 17.54], *P* = 0.02).

**Table 3:** Univariable analysis of time to progression of True low‐grade dysplasia to high‐grade dysplasia/esophageal adenocarcinoma

Variable		Progression	Hazard ratio (95% confidence interval)	*P* value
Age^‡^	—	—	1.00 (0.96, 1.04)	0.99
Gender	Female	¼	1	0.98
	Male	13/46	1.03 (0.13, 7.97)	
Nodularity	No	9/39	1	0.03
	Yes	5/11	3.56 (1.13, 11.27)	
Location	Unifocal LGD	3/25	1	0.02
	Multifocal LGD	11/23	4.82 (1.33, 17.54)	
Hiatus hernia (HH)	No	6/22	1	0.83
	Yes	8/28	0.89 (0.31, 2.59)	
HH size^§^	Small (<3 cm)	5/13	1	0.60
	Large (>3 cm)	3/15	0.68 (0.16, 2.88)	
Length (C)^†^	—	—	1.01 (0.90, 1.15)	0.82
Length (M)^†^	—	—	0.98 (0.85, 1.14)	0.83
Smoking status	Non‐smoker	7/21	1	0.72
	Current smoker	1/7	1.35 (0.28, 6.49)	
	Ex‐smoker	2/7	0.58 (0.05, 6.47)	
PPI medication	No	1/3	1	0.50
	Yes	12/40	2.04 (0.26, 16.19)	
Endoscopic therapy during follow up and after referral	No	11/33	1	0.09
	Yes	3/17	0.32 (0.09, 1.18)	

^†^ Hazard ratios given for a 1‐unit increase in variable.

^‡^ Hazard ratios given for a 1‐unit increase in variable.

^§^ Analysis for patients with hiatus hernia only.

Multivariable analysis suggested some evidence that nodularity at index endoscopy, location of low‐grade dysplasia, and endoscopic therapy were associated with time to progression (Table 4). Patients undergoing endoscopic therapy had a three times lower risk of progression compared with patient who never underwent endoscopic therapy and just undertook surveillance follow‐up endoscopies. The risk of progression at any time was six times higher for patients with nodularity at index endoscopy compared with patients without, while the risk of progression was almost four times higher in multifocal LGD compared with unifocal LGD.

**Table 4:** Multivariable analysis of time to progression of T‐LGD to HGD/EAC

Variable	Category	Hazard ratio (95% confidence interval)	*P* value
Nodularity at index endoscopy	No Yes	1 5.54 (1.60, 19.17)	0.007
Location of dysplasia	Unifocal LGD Multifocal LGD	1 3.78 (0.98, 14.59)	0.05
Endoscopic therapy	No Yes	1 0.31 (0.08, 1.22)	0.09

EAC, esophageal adenocarcinoma; HGD, high‐grade dysplasia; T‐LGD, true low‐grade dysplasia.

### *Comparison of the T‐LGD* versus *the DS‐LGD‐NDBE/DS‐LGD‐IND cohort*

**Figure 2:**
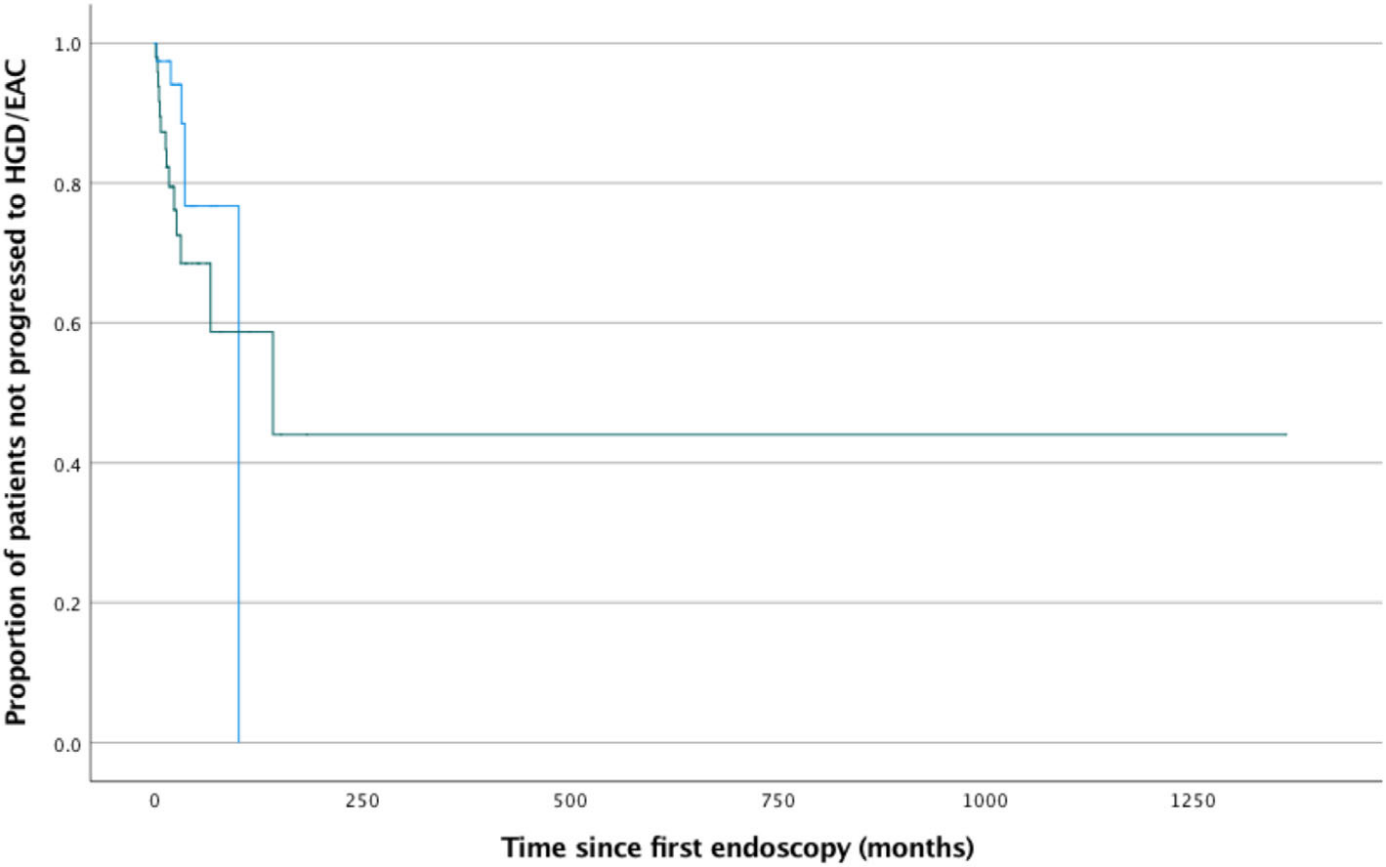
Kaplan–Meier plot showing time to progression of T‐LGD (*n* = 50) *versus* DS‐LGD‐NDBE/DS‐LGD‐IND (*n* = 41) to high‐grade dysplasia/esophageal adenocarcinoma. There was no significant difference (*P* = 0.21). Time 0 represents the start of the follow‐up period. DS‐LGD‐IND, low‐grade dysplasia downstaged to indefinite for dysplasia; DS‐LGD‐NDBE, low‐grade dysplasia downstaged to non‐dysplastic Barrett's esophagus; EAC, esophageal adenocarcinoma; HGD, high‐grade dysplasia; T‐LGD, true low‐grade dysplasia. 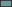, T‐LGD group; 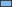, downstaged group (DS‐LGD‐NDBE/DS‐LGD‐IND).

Sixty‐nine percent of patients in the T‐LGD cohort had not progressed to HGD/EAC at 5 years. Overall 14 of 50 patients with T‐LGD progressed to HGD/EAC. Seventy‐seven percent of patients in the DS‐LGD‐NDBE/DS‐LGD‐IND cohort did not progress to HGD/EAC at 5 years. Six of 41 patients with DS‐LGD‐NDBE/DS‐LGD‐IND progressed overall. There was no significant difference in time to progression between patients with T‐LGD and those who were downstaged to IND/NDBE (*P* = 0.21) (Fig. 2).

### Natural history of progression of Barrett's low‐grade dysplasia

Thirty‐three patients had T‐LGD diagnosed by two expert histopathologists and no prospective history of ablation therapy. Fifty‐nine percent of these patients had not progressed to HGD/EAC at 5 years. Overall, 11 of 33 patients progressed to HGD/EAC. The median time to progression was 67 months (95% CI: 3–131) (Table 5).

**Table 5:** Natural history of progression in patients with no history of ablation therapy

	T‐LGD (*n* = 33)	DS‐LGD‐IND (*n* = 15)	DS‐LGD‐NDBE (*n* = 20)
Mean age, years	72	71	71
Male sex	31/33 (94%)	13/15 (87%)	16/20 (80%)
Proportion of patients that have not progressed at 5 years	59%	78%	74%
Number of patients progressing to HGD/EAC	11	2	3

DS‐LGD‐IND, low‐grade dysplasia downstaged to indefinite for dysplasia; DS‐LGD‐NDBE, low‐grade dysplasia downstaged to non‐dysplastic Barrett's esophagus; EAC, esophageal adenocarcinoma; HGD, high‐grade dysplasia; T‐LGD, true low‐grade dysplasia.

Fifteen patients were downgraded to IND and had no prospective history of ablation therapy treatment. Seventy‐eight percent of these patients had not progressed to HGD/EAC at 5 years. Two of 15 patients overall progressed to HGD/EAC. Twenty patients were downgraded to NDBE and had no prospective history of ablation therapy. Seventy‐four percent of these patients had not progressed to HGD/EAC at 5 years. Three of 20 patients progressed overall to HGD/EAC.

The overall median time to progression in these three cohorts was 101 months (95% CI: 52–150). There was no significant difference in the time to progression between the three cohorts (*P* = 0.22).

## Discussion

Barrett's dysplasia is associated with an increased risk of progression to EAC/HGD. Guidance regarding the management of EAC/HGD in BE is clear in terms of offering endoscopic eradication therapy as a first‐line treatment. The management of patients with LGD can include either surveillance endoscopies with biopsies or endoscopic eradication therapy.^11^, ^17^, ^18^ A particular issue is the variation in the diagnosis of LGD, which can contribute to concerns in offering endoscopic therapy given the potential risks.

Endoscopic eradication therapy is safe and effective for the treatment of LGD‐BE but comes at a patient cost. The SURF study showed that 12% of patients developed a stricture after RFA requiring endoscopic dilatation and three serious adverse events were observed.^7^ There were no adverse events in the surveillance cohort. We need to not over treat this patient cohort.

In this study, there is clear variability in the diagnosis of LGD from referring centers. Only a third of all patients had confirmed LGD following review by two expert histopathologists. Thirty‐two percent of patients were upstaged to HGD and a third of patients were DS‐LGD‐NDBE/DS‐LGD‐IND and continued surveillance endoscopies with biopsies. This reaffirms the importance of the requirement of a diagnosis of LGD to be reviewed and confirmed by two expert pathologists. A study found excellent concordance between histopathologists in the diagnosis of HGD and NDBE (>70%), however intermediate agreement for LGD among 51 pathologists (42%).^18^ A study found that 73% of patients with LGD in BE had their diagnosis downstaged to NDBE/IND and they had a lower risk of progression compared with the T‐LGD cohort.^19^

The cumulative incidence of progression to HGD/EAC and time to progression varied across subgroups. The T‐LGD cohort of patients had double the rates of progression compared with the downstaged cohort. At 5 years, 59% of the T‐LGD cohort of patients had not progressed *versus* 78% and 74% in the DS‐LGD‐IND and DS‐LGD‐NDBE cohort, respectively. This suggests that this is a particularly high‐risk cohort of patients with a higher risk of progression over a shorter period. It is important to differentiate the patient subgroups. Decisions on surveillance and endotherapy can be more personalized and resources utilized more wisely. A previous study of 147 patients diagnosed with LGD showed that patients with T‐LGD had a cumulative risk of progression of 85% in 109.1 months, relative to 4.6% in 107.4 months for the DS‐LGD‐NDBE/DS‐LGD‐IND cohort.^20^

Five out of 35 patients in the ablation naïve cohort who were downstaged to NDBE/IND progressed to HGD/EAC. This is a much smaller proportion compared with 11 of 33 patients who progressed in the true LGD cohort. The proportion of patients who progressed in the downstaged cohort was higher than a previous study where the 5‐year cumulative risk of progression was 2.9% and 2.1% in the downstaged IND and NDBE cohort.^19^ This reflects the variability in the diagnosis of LGD, and these five patients in our study may have been downstaged but in reality they may have had true LGD. The other important reason for this difference may have been that there were a smaller number of patients in the ablation naïve cohorts in our study, therefore it would be difficult to do a direct comparison. Given the risk of progression in the downstaged cohort, an argument can be made for ablation treatment in this cohort; however, this does carry risks and the majority of patients in this group do not progress. An alternative would be an adjusted shorter surveillance interval for these group of patients who do not carry the same progression risk as the true low‐grade patients. An alternative strategy would be increasing the number of pathologists reviewing the histology slides of patients with low‐grade dysplasia.

In our study, patients with LGD who had ablative therapy had a three times lower risk of progression relative to patients who were followed up with long‐term surveillance biopsies. The long‐term outcomes of a randomized control trial showed that RFA in LGD significantly reduces the risk of progression after a median follow up of 73 months.^21^ Our study affirms the importance in offering endoscopic therapy to the T‐LGD cohort. They are a particularly higher‐risk cohort. A study found that patients with LGD in BE, who were treated with ablation, reported a quality of life comparable with patients undergoing endoscopic surveillance.^22^ In our study, patients received either RFA, cryoablation, or argon plasma coagulation therapy.

Thirty‐three patients had T‐LGD and no ablation history allowing us to analyze their natural history. Seventy percent of patients were diagnosed prior to the updated 2015 BSG guidelines. At that point the recommendation for LGD in BE was a repeat endoscopy every 6 months.

A study carried out an analysis from three population‐based models showing the optimal management for patients with LGD in BE is endoscopic eradication therapy only after LGD is confirmed.^23^ These are findings confirmed in our study where all patients had a second endoscopy to confirm LGD given the variability in its diagnosis with a reduction in rates of progression in patients receiving ablation therapy. This allows therapy to be focused on the T‐LGD patients (38% of the overall patient cohort).

The two main risk factors for progression in our cohort of patients were the presence of nodularity at index endoscopy and multifocal LGD (*P* < 0.05). If the nodularity was unifocal or multifocal did not have an effect on outcomes. Ninety‐five percent of all patients who progressed were male. Age, gender, Barrett's length, and smoking history were not associated with risk of progression. There have been variations in risks for progression in different studies. A multicenter prospective registry study showed that the risk of progression to HGD/EAC was eight‐fold higher in the patient cohort where two expert gastrointestinal pathologists re‐confirmed a diagnosis of LGD.^9^ Another multicenter study showed that there were no risk factors for progression with significant interobserver variation in diagnosis among expert pathologists.^10^ A single‐center retrospective study of 69 patients showed that persistent LGD was an independent risk factor for progression to HGD/EAC.^11^ Khan et al. showed that the length of BE was associated with risk of progression.^24^ Identifying key risk factors of progression will allow the building of a risk stratification tool, which will help tailor treatment in a specific, higher‐risk cohort.

There are some limitations to the study. This is a single‐center study and data collection was done retrospectively. In future work, we will include an increased variability of pathologists to review histopathology slides to reach a global consensus regarding the diagnosis of low‐grade dysplasia. This will allow us to investigate the variability in diagnosis and further confirm the difficulty in diagnosing these cohort of patients where there needs to be greater consensus in pathological criteria for diagnosis. There may be further variation in the number of patients downstaged to IND/NDBE and number of patients with T‐LGD.

The outcomes of our study suggest there needs to be more stringent pathological criteria for the diagnosis of LGD in BE in the community. The T‐LGD cohort is a high‐risk cohort, and these patients need to be identified and if fit they should undergo endoscopic therapy following patient discussion. Certain variables can be used to identify those much higher‐risk patients. The presence of nodularity at index endoscopy and multifocal LGD seems to be associated with higher progression rates. A risk stratification tool will help identify high‐risk LGD patients who require endoscopic eradication therapy.

## References

[1] Vennalaganti P, Kanakadandi V, Goldblum J*et al*. Discordance among pathologists in the United States and Europe in diagnosis of low‐grade dysplasia for patients with Barrett's esophagus. Gastroenterology. 2017; 152: 564–70. 27818167 10.1053/j.gastro.2016.10.041

[2] Solanky D, Krishnamoorthi R, Crews N*et al*. Barrett esophagus length, nodularity, and low‐grade dysplasia are predictive of progression to esophageal adenocarcinoma. J. Clin. Gastroenterol. 2019; 53: 361–5. 29608452 10.1097/MCG.0000000000001027

[3] Kambhampati S, Tieu AH, Luber B, Wang H, Meltzer SJ. Risk factors for progression of Barrett's esophagus to high grade dysplasia and esophageal adenocarcinoma. Sci. Rep. 2020; 10: 4899. 32184470 10.1038/s41598-020-61874-7 PMC7078316

[4] O'Byrne LM, Witherspoon J, Verhage RJJ*et al*. Barrett's registry collaboration of academic centres in Ireland reveals high progression rate of low‐grade dysplasia and low risk from nondysplastic Barrett's esophagus: report of the RIBBON network. Dis esophagus. 2020; 33: doaa009. 32193532 10.1093/dote/doaa009

[5] Wani S, Rubenstein J, Vieth M, Bergman J. Diagnosis and management of low‐grade dysplasia in Barrett's esophagus: expert review from the clinical practice updates committee of the American Gastroenterological Association. Gastroenterology. 2016; 151: 822–35. 27702561 10.1053/j.gastro.2016.09.040

[6] Qumseya BJ, Wani S, Gendy S, Harnke B, Bergman JJ, Wolfsen H. Disease progression in Barrett's low‐grade dysplasia with radiofrequency ablation compared with surveillance: systematic review and meta‐analysis. Am. J. Gastroenterol. 2017; 112: 849–65. 28374819 10.1038/ajg.2017.70

[7] Phoa KN, van Vilsteren FGI, Weustan BLAM*et al*. Radiofrequency ablation vs endoscopic surveillance for patients with Barrett esophagus and low‐grade dysplasia. JAMA. 2014; 311: 1209–17. 24668102 10.1001/jama.2014.2511

[8] Skacel M, Petras RE, Gramlich TL, Sigel JE, Richter JE, Goldblum JR. The diagnosis of low‐grade dysplasia in Barrett's esophagus and its implications for disease progression. Am. J. Gastroenterol. 2000; 95: 3383–7. 11151865 10.1111/j.1572-0241.2000.03348.x

[9] Krishnamoorthi R, Lewis JT, Krishna M*et al*. Predictors of progression in Barrett's esophagus with low‐grade dysplasia: results from a multicentre prospective BE registry. Am. J. Gastroenterol. 2017; 112: 867–73. 28374813 10.1038/ajg.2017.84

[10] Wani S, Falk GW, Post J*et al*. Risk factors for progression of low‐grade dysplasia in patients with Barrett's esophagus. Gastroenterology. 2011; 141: 1179–86. 21723218 10.1053/j.gastro.2011.06.055

[11] Song KY, Henn AJ, Gravely AA*et al*. Persistent confirmed low‐grade dysplasia in Barrett's esophagus is a risk factor for progression to high‐grade dysplasia and adenocarcinoma in a US veterans cohort. Dis. Esophagus. 2020; 33: doz061. 31274147 10.1093/dote/doz061

[12] Falk GW. Current management of low‐grade dysplasia in Barrett esophagus. Gastroenterol. Hepatol. 2017; 13: 221–5. PMC5441023 28546793

[13] Spechler SJ, Sharma P, Souza RF, Inadomi JM, Shaheen NJ. American Gastroenterological association medical position statement on the management of Barrett esophagus. Gastroenterology. 2011; 140: 1084–91. 21376940 10.1053/j.gastro.2011.01.030

[14] Evans JA, Early DS, Fukami N, ASGE standards of practice committee; standards of practice committee of the American society for gastrointestinal endoscopy . The role of endoscopy in Barrett's esophagus and other premalignant conditions of the esophagus. Gastrointest. Endosc. 2012; 76: 1087–94. 23164510 10.1016/j.gie.2012.08.004

[15] Fitzgerald RC, di Pietro M, Ragunath K*et al*. British society of gastroenterology. British society of gastroenterology guidelines on the diagnosis and management of Barrett's oesophagus. Gut. 2014; 63: 7–42. 24165758 10.1136/gutjnl-2013-305372

[16] Weustan B, Bisschops R, Coron E*et al*. Endoscopic management of Barrett's esophagus: European Society of Gastrointestinal Endoscopy (ESGE) position statement. Endoscopy. 2017; 49: 191–8. 28122386 10.1055/s-0042-122140

[17] Shaheen NJ, Falk GW, Iyer P, Gerson LB. ACG clinical guideline: diagnoses and management of Barrett's esophagus. Am. J. Gastroenterol. 2016; 111: 30–50. 26526079 10.1038/ajg.2015.322 PMC10245082

[18] Van der Wel MJ, Coleman HG, Bergman JJGHM, Jansen M, Meijer SL. Histopathologist features predictive of diagnostic concordance at expert level among a large international sample of pathologists diagnosing Barrett's dysplasia using digital pathology. Gut. 2020; 69: 811–22. 31852770 10.1136/gutjnl-2019-318985

[19] Duits L, Phoa KN, Curvers WL*et al*. Barrett's oesophagus with low‐grade dysplasia can be accurately risk‐stratified after histological review by an expert pathology panel. Gut. 2015; 65: 700–6. 10.1136/gutjnl-2014-307278 25034523

[20] Curvers WL, Kate FJT, Krishnadath KK*et al*. Low‐grade dysplasia in Barrett's esophagus: overdiagnosed and underestimated. Am. J. Gastroenterol. 2010; 105: 1523–30. 20461069 10.1038/ajg.2010.171

[21] Pouw RE, Klaver E, Phoa KN*et al*. Radiofrequency ablation for low‐grade dysplasia in Barrett's esophagus: long‐term outcome of a randomized trial. Gastrointes. Endosc. 2020; 92: 569–74. 10.1016/j.gie.2020.03.3756 32217112

[22] Rosmolen WD, Phoa NKY, Nieuwkerk PT*et al*. Impact of ablation of Barrett's esophagus with low‐grade dysplasia on patients' illness perception and quality of life: a multicentre randomized trial. Gastrointest. Endosc. 2019; 90: 215–21. 31026438 10.1016/j.gie.2019.04.226

[23] Omidvari AH, Ali A, Hazelton WD*et al*. Optimizing management of patients with Barrett's esophagus and low‐grade or no dysplasia based on comparative modelling. Clin. Gastroenterol. Hepatol. 2020; 18: 1961–9. 31816445 10.1016/j.cgh.2019.11.058 PMC7447845

[24] Khan A, Kommineni VT, Callaway JK*et al*. 344 outcomes of radiofrequency ablation and risk for dysplastic progression in Barrett's esophagus with low‐grade dysplasia: experience at a tertiary care centre academic medical center. Gastrointest Endosc. 2015; 81: AB140–1 (Abstract).

